# Food worry and mental health outcomes during the COVID-19 pandemic

**DOI:** 10.1186/s12889-022-13410-7

**Published:** 2022-05-17

**Authors:** Brenna B. Han, Eva Purkey, Colleen M. Davison, Autumn Watson, Dionne Nolan, Dan Mitchell, Sheldon Traviss, Jennifer Kehoe, Imaan Bayoumi

**Affiliations:** 1 Queen’s School of Medicine, 15 Arch St, Kingston, ON K7L 3L4 Canada; 2 Department of Family Medicine, 220 Bagot St, Kingston, ON K7L 3G2 Canada; 3 Centre for Studies in Primary Care, 220 Bagot St, Kingston, ON K7L 3G2 Canada; 4 Queen’s University Public Health Sciences, 62 Fifth Field Company Lane, Kingston, ON K7L 3N6 Canada; 5 Indigenous Diabetes Health Circle, 3250 Schmon Pkwy, Thorold, ON L2V 4Y6 Canada; 6 Indigenous Health Council, Kingston, ON Canada

**Keywords:** Food insecurity, Mental health, COVID-19 pandemic

## Abstract

**Background:**

There is limited and inconsistent literature examining the relationship between food worry and mental health in the context of the COVID-19 pandemic. This study examined the association between food worry and mental health among community dwelling Canadian adults during the COVID-19 pandemic.

**Methods:**

Adults age 16 years and older completed an anonymous online questionnaire between April 1, 2020 and November 30 2020. Measures of pre-pandemic and current food worry, depression (PHQ-2), anxiety (GAD-2), and sociodemographic variables were included. Multivariable logistic regression models were used to determine the association between food worry and symptoms of depression and anxiety.

**Results:**

In total, 1605 participants were included in analyses. Worry about affording food was reported by 320 (14.78%) participants. In models adjusting for sociodemographic covariates, compared with people without food worry, participants who had food worry were 2.07 times more likely to report anxiety symptoms (aOR 2.07, 95% CI: 1.43 – 2.98, *p* < .001) and were 1.9 times more likely to report depressive symptoms (aOR 1.89, 95% CI: 1.39–2.57, *p* < .0001). Lower income, lower education, and pre-existing mental health conditions were significant predictors of symptoms of depression. Female gender, younger age, lower education, lower income, and pre-existing mental health condition were significant predictors of anxiety symptoms.

**Conclusion:**

Our study highlights the relationship between food worry and poor mental health. Policy supports such as improved income supports, clinical implications such as screening for food worry in primary care, referral to emergency food programs and support with meal planning may help mitigate mental health symptoms during the current pandemic, during future societal recovery from this pandemic and during future pandemics.

**Supplementary Information:**

The online version contains supplementary material available at 10.1186/s12889-022-13410-7.

## Introduction

The COVID-19 pandemic has altered daily life in Canadian households, particularly on matters of financial and food security. Households have encountered pandemic-related economic contraction, job losses, supply chain disruptions, and rising food costs [1]. In April 2020, 24% of Canadian adults indicated fair or poor mental health [2]. In comparison, only 8% of Canadians reported fair or poor mental health in the 2018 Canadian Community Health Survey. Pandemic-related fears about health for oneself and loved ones, prolonged social isolation, and feelings of uncertainty were common sources of distress. One source of distress during this time may be worry about the ability to afford food, especially as food insecurity has increased. A May 2020 survey found pandemic-era prevalence of food insecurity increased to 14.6% compared to the 2017–2018 Canadian Community Health Survey results (10.5%) [2]. Even prior to the pandemic, food insecurity disproportionately affected Indigenous households—where almost three in ten Indigenous adults lived in food-insecure households [3]. The arrival of COVID-19 further exacerbated already existing food security challenges faced by Indigenous communities, with a high levels of economic, health, and social inequities [4].

Food security can be classified into four categories using the Household Food Security Module, a validated measure with categories determined by responses to specific questions: food secure, marginal food insecurity, moderate food insecurity, and severe food insecurity [5, 6]. The concept of *food worry*, the experience of stress or worry about having enough food to meet basic needs [7], is connected most closely with the classification of marginal food insecurity, which is described as being worried that food would run out before getting money to buy more [5]. In contrast, there is a compromise in the quality and/or quantity of food consumed by the household in more severe forms of food insecurity. Prior research has identified that households experiencing marginal food insecurity are more similar to households experiencing moderate to severe food insecurity in terms of household sociodemographic characteristics than to food secure households [8].

Previous literature has demonstrated the association between food insecurity and poor mental health. Food-insecure individuals are at increased risk for depression and anxiety symptoms and diagnoses [9–12]. While the associations between household food insecurity and adverse mental health outcomes are observed along a severity gradient, where more severe food insecurity is associated with a poorer mental health outcomes such as depressive symptoms and suicidal thoughts, marginal food insecurity was still associated with significantly higher odds of poor mental health [13–15]. In addition, low household income is associated with both food insecurity [16] and with poorer mental health [17].

Exploration of the association between food insecurity and mental health during the COVID-19 pandemic has been limited, with inconsistent findings [18, 19] and only one study has examined food worry specifically [7]. No previous literature has examined the association between food insecurity and mental health during previous pandemics. This study assessed the association between financially related food worry and symptoms of anxiety and depression during the COVID-19 pandemic.

## Materials and methods

The *Cost of COVID* study examined the social and emotional impacts of COVID-19 pandemic-related public health measures with a sub-focus on urban Indigenous People. Data were collected from April 1, 2020 to November 30, 2020. The majority of recruitment occurred in the Kingston, Ontario, Canada region.

Consenting adults age 16 or older were invited to complete an anonymous online survey. Recruitment was conducted virtually, using advertisements on social media platforms (Facebook, Twitter, and Instagram). Additionally, local service organizations were asked to disseminate the survey. There was targeted recruitment among urban Indigenous People and Indigenous service organizations were prioritized for recruitment. The study was approved by the Queen’s University Health Sciences Research Ethics Board.

To ensure effective and respectful data collection and analysis, the First Nations principles of Ownership, Control, Access, and Possession (OCAP®) were applied throughout the entire research process. The OCAP® principles represent a set of standards that establish how First Nations data should be collected, protected, used, and shared (i.e. the standard for how to conduct research with First Nations people). These principles assert that First Nations people have control over data collection processes in their communities, and that they own and control how this information can be used; OCAP® is a registered trademark of the First Nations Information Governance Centre [20]. To adhere to these principles, our team included an Indigenous research associate (AW) and consulted regularly with a sub-committee of the local Indigenous Health Council (DN, JK, DM, ST) in order to operationalize OCAP®. The intention of these meetings was to discuss and review research findings, and to support the interpretation of results and knowledge translation.

### Data collection

The anonymous online questionnaire was administered using Qualtrics^XM^ survey software, and included measures of mental health, income, and financial strain, among others.

#### Food worry

Participants were asked to indicate their level of worry about their ability to pay for food in the past 2 weeks and before March 2020. They were asked “How worried have you been about running out of food and not having enough money to buy more?”, with response options: very worried, somewhat worried, a little worried, not very worried, not at all worried.

Presence of financially related food worry was categorized by including response options “very worried”, “somewhat worried”, and “a little worried”.

#### Mental health

To assess participants’ mental health at the time of participation, the two-item Patient Health Questionnaire (PHQ-2) and the Generalized Anxiety Disorder (GAD-2) were used. For both scales, participants with a score of 3 or more were considered a positive screen for symptoms of depression or anxiety. The PHQ-2 is a validated tool to screen for symptoms of depression [21–23] and assesses the severity of depressive symptoms over the past 2 weeks: “Over the last 2 weeks, how often have you been bothered by any of the following problems?”. The two items are “Little interest or pleasure in doing things” and “Feeling down, depressed, or hopeless”. Respondents can rate their response from 0 (not at all) to 3 (nearly every day). The total score ranges from 0 – 6, where a score of 3 is the validated cut point when screening for depression. The PHQ-2 has been shown to have good reliability in terms of internal consistency (α = 0.84) and intraclass correlation scores (ICC = 0.92) in international studies [24–27].

The GAD-2 is a validated tool to screen for symptoms of anxiety [28], which uses two questions to measure the severity of anxiety symptoms in the last 2 weeks: “Over the last 2 weeks, how often have you been bothered by any of the following problems?”. The two items are “Feeling nervous, anxious or on edge?” and “Not being about to stop or control worrying?”. Like the PHQ-2, responses range from 0 (not at all) to 3 (nearly every day). A score of 3 is the cut point for screening for anxiety. Studies examining the test properties of the GAD-2 reported a pooled sensitivity of 0.76 (95% CI 0.55–0.89), and specificity of 0.81 (95% CI 0.60–0.92) compared with gold standard measures [28–30].

#### Demographic variables

Participants were asked to indicate their age, gender, ethnicity, education, current employment status, and estimated annual household income after tax the previous year.

### Data analysis

Survey data were analyzed using SAS OnDemand for Academics [31]. Descriptive statistics were determined, including counts and proportions for categorical variables and means and standard deviations for continuous variables.

We conducted unadjusted and adjusted logistic regression analyses to examine the association between food worry and symptoms of depression and anxiety. We adjusted for the following covariates: gender, age, self-reported household income, self-reported ethnicity, highest level of education completed, past food worry, and pre-existing mental health condition. We used complete cases in our analysis. Variables used in modelling were missing between 0–8%, with the exception of ethnicity which was missing among 17.3% of respondents. The final sample included in the multivariate models was 1605 participants. To better understand the impact of missing data, we conducted a sensitivity analysis using multiple imputation using the fully conditional specification method to impute missing covariates. To reduce the potential for bias, models were run on 20 imputed data sets using all identified covariates. Results of the 20 imputed data sets were combined, and the parameter estimates (95%CIs) for the adjusted pooled models were reported.

## Results

**Fig. 1:**
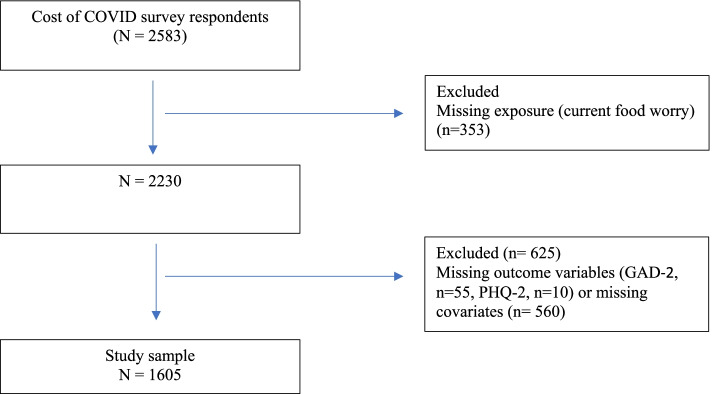
Flow diagram of the study inclusion/exclusion process

The Cost of COVID Study enrolled 2583 participants, including 2165 participants with complete exposure and outcome data (study flowchart seen in Fig. 1). The sample characteristics are reported in Table 1. The sample was primarily female (87.9%), of European descent (62.0%), well educated (44.48% had obtained a university degree), and younger than 50 years of age (76.8%). The sample included 241 respondents (11.1%) who identified Indigenous ancestry.

**Table 1:** Characteristics of participants (*n* = 2165)

Variable	Food Worry *n* = 320 N (%), Median (IQR)	No Food Worry *n* = 1845 N (%), Median (IQR)	Total *n* = 2165 N (%), Mean (SD)
**Gender**			
Male	27 (8.44)	206 (11.21)	233 (10.77)
Female	283 (88.4)	1611 (87.65)	1901 (87.89)
Non-binary^a^	10 (3.13)	18 (0.98)	28 (1.29)
Missing	0 (0)	3 (0.16)	3 (0.14)
**Age**			
16–34 years	156 (48.75)	795 (43.25)	951 (43.93)
35–49 years	107 (33.44)	601 (32.70)	708 (32.70)
50–64 years	51 (15.94)	301 (16.38)	352 (16.26)
≥ 65 years	6 (1.88)	141 (7.67)	287 (13.26)
**Missing**	0	0	0
**Ethnicity**			
Indigenous	61 (25.21)	180 (9.76)	241 (11.14)
Asian, Middle Eastern, African, Latin	33 (13.64)	175 (9.49)	208 (9.62)
American			
European	148 (61.16)	1194 (64.72)	1342 (62.04)
Missing	78 (0.24)	296 (16.04)	374 (17.29)
**Household Income**			
< $30,000	181 (56.56)	362 (19.62)	543 (25.01)
$30,000 -$79,999	74 (23.12)	481 (26.07)	555 (25.64)
$80,000-$149,999	51 (15.94)	662 (35.88)	713 (32.93)
≥ $150,000	8 (2.50)	287 (15.56)	295 (13.63)
Missing	6 (1.88)	52 (2.82)	58 (2.68)
**Education**^b^			
High School or Less, other	143 (44.69)	498 (26.99)	641 (29.63)
College diploma	120 (37.50)	440 (23.85)	560 (25.89)
University degree	57 (17.81)	905 (49.05)	962 (44.48)
Missing	0 (0)	2 (0.11)	2 (0.09)
**Pre-existing Mental Health Condition**			
No	118 (36.88)	1088 (58.97)	1206 (55.70)
Yes	180 (56.25)	623 (33.77)	803 (37.09)
Missing	22 (6.88)	134 (7.26)	156 (7.21)
**Employment**			
Employed, not specified	10 (3.13)	111 (6.01)	121 (5.59)
Employed full-time	70 (21.88)	809 (43.85)	879 (40.60)
Employed part-time	40 (12.50)	216 (11.71)	256 (11.82)
Unemployed	173 (54.06)	609 (33.01)	782 (36.12)
Missing	27 (8.44)	100 (5.42)	127 (5.87)
PHQ	3.00 (2.00)	2.00 (3.00)	2.00 (1.77)
PHQ ≥ 3	164 (51.25)	473 (25.64)	637 (29.42)
GAD	4.00 (3.50))	2.00 (2.00)	2.40 (1.84)
GAD ≥ 3	195 (60.94)	577 (31.27)	772 (35.66)

^a^Transgender, gender fluid, non-binary, Two-Spirit

^b^Highest level education completed

Current food worry was reported by 320 (14.8%) participants. Participants with food worry were more likely than those without food worry to self-identify as having Indigenous ancestry, have lower levels of completed education (high school education or less), be unemployed, and report household income below $30,000/year. Compared to those without food worry, individuals with food worry reported higher mean scores for the PHQ-2 (3.03 vs 1.82) and GAD-2 (3.53 vs. 2.21). The prevalence of food worry was more than three times higher among Indigenous participants compared to non-Indigenous participants (34.2% vs 13.1%). Indigenous participants also reported lower incomes (34.7% reported household income < $30,000) compared with those of European ancestry (21.2%). Similar results were observed regarding education (16.1% of Indigenous respondents reported their highest level of education was high school, compared with 24.4% of those of European ancestry.

In adjusted models reported in Tables 2 and 3, food worry was significantly associated with both depression and anxiety. Participants who had food worry had a 2.07 greater odds of reporting symptoms of anxiety (95% CI: 1.43 – 2.98, *p* < 0.0001) (Table 2) and 1.89 times greater odds of reporting depressive symptoms (95% CI: 1.32 – 2.71, *p* = 0.0006) (Table 3) compared with those without food worry. Lower income, lower education, and pre-existing mental health conditions were statistically significant predictors of symptoms of depression (Table 3). Female gender, younger age (less than 50 years of age), lower education, lower income, and pre-existing mental health condition were statistically significant predictors of anxiety symptoms (Table 2).

**Table 2:** Univariate and multivariate logistic regression model examining the association of food worry and symptoms of anxiety (GAD ≥ 3) *n* = 1605

Variables	Univariate regression		Multivariate regression	
	**OR (95% CI)**	***p***	**aOR (95% CI)**	***p***
Current food worry	3.43 (2.68 – 4.38)	< .0001	2.07 (1.43 – 2.98)	< .0001
No current food worry	Reference		Reference	
Past food worry	3.20 (2.32 – 4.42)	< .0001	1.17 (0.74 – 1.87)	0.50
No past food worry	Reference		Reference	
**Gender**				
Female	1.20 (1.45 – 2.75)	< 0.001	1.74 (1.17 – 2.59)	0.006
Male	Reference		Reference	
Non-binary^a^	3.92 (1.76 – 8.75)	< 0.001	1.89 (0.66 – 5.42)	0.2386
**Ethnicity**				
Indigenous	1.58 (1.20 – 2.09)	< 0.01	0.97 (0.69 – 1.36)	0.86
Asian, Middle Eastern, African, Latin American	1.15 (0.85 – 1.55)	0.38	1.02 (0.70 – 1.47)	0.93
European	Reference		Reference	
**Age**				
16 – 34 years	2.13 (1.63 – 2.79)	< .0001	2.00 (1.40 – 2.84)	0.0001
35 – 49 years	1.50 (1.13 – 1.99)	< 0.01	1.63 (1.13 – 2.34)	0.009
50 – 64 years	Reference		Reference	
≥ 65 years	0.29 (0.16 – 0.52)	< .0001	0.30 (0.14 – 0.66)	0.002
**Household income**				
< $30,000	2.63 (1.94 – 3.56)	< .0001	1.84 (1.28 – 2.64)	0.001
$30,000 – 79,999	1.15 (0.84 – 1.56)	0.38	1.10 (0.77 – 1.56)	0.61
80,000 – 149,000	1.05 (0.78 – 1.42)	0.74	1.05 (0.75 – 1.47)	0.77
≥ $150,000	Reference		Reference	
**Education**^b^				
High school or less	2.11 (1.71 – 2.60)	< .0001	1.56 (1.17 – 2.07)	0.003
College diploma	1.68 (1.35 – 2.10)	< .0001	1.14 (0.85 – 1.54)	0.37
University	Reference		Reference	
**Pre-existing mental health condition**				
Yes	4.60 (3.79 – 5.59)	< .0001	3.81 (3.02 – 4.82)	< .0001
No	Reference		Reference	

^a^Transgender, gender fluid, non-binary, Two-Spirit

^b^highest completed

Model adjusted for gender, ethnicity, age, income, education, past food worry and pre-existing mental health condition

**Table 3:** Univariate and multivariate logistic regression models examining the association of food worry and symptoms of depression (PHQ ≥ 3) *n* = 1605

Variables	Univariate regression		Multivariate regression	
	**OR (95% CI)**	***p***	**aOR (95% CI)**	***p***
Current food worry	3.05 (2.39 – 3.89)	< .0001	1. 89 (1.39 – 2.57)	0.0006
No current food worry	Reference		Reference	
Past food worry	3.03 (2.20 – 4.16)	< .0001	0.96 (0.60 – 1.51)	0.84
No past food worry	Reference		Reference	
**Gender**				
Female	1.68 (1.20 – 2.34)	< 0.01	1.46 (0.97 – 2.19)	0.07
Male	Reference		Reference	
Non-binary^a^	3.34 (1.49 – 7.49)	< 0.01	0.86 (0.30 – 2.45)	0.77
**Ethnicity**				
Indigenous	1.81 (1.37 – 2.41)	< .0001	1.17 (0.84 – 1.64)	0.35
Asian, Middle Eastern, African, Latin American	1.12 (0.82 – 1.55)	0.48	1.15 (0.78 – 1.69)	0.47
European	Reference		Reference	
**Age**				
16 – 34 years old	1.61 (1.22 – 2.12)	< 0.001	1.40 (0.97 – 2.01)	0.07
35 – 49 years old	1.10 (0.82 – 1.48)	0.51	1.15 (0.79 – 1.68)	0.47
50 – 64 years old	Reference		Reference	
≥ 65 years old	0.73 (0.46 – 1.17)	0.19	0.77 (0.41 – 1.44)	0.42
**Household income**				
< $30,000	3.04 (2.19 – 4.22)	< .0001	1.67 (1.09 – 2.56)	0.02
$30,000 – $79,999	1.40 (0.99 – 1.96)	0.05	1.07 (0.71 – 1.61)	0.76
$80,000 – $149,000	1.20 (0.86 – 1.67)	0.28	1.02 (0.68 – 1.51)	0.94
≥ $150,000	Reference		Reference	
**Education**^b^				
High school or less	2.53 (2.03 – 3.16)	< .0001	1.92 (1.43 – 2.58)	< .0001
College	1.89 (1.49 – 2.39)	< .0001	1.43 (1.05 – 1.94)	0.02
University	Reference		Reference	
**Pre-existing mental health condition**				
Yes	5.06 (4.12 – 6.21)	< .0001	4.04 (3.17 – 5.14)	< .0001
No	Reference		Reference	

^a^Transgender, gender fluid, non-binary, Two-Spirit

^b^Highest level education completed

Model adjusted for gender, ethnicity, age, income, education, past food worry and pre-existing mental health condition

We then used multiple imputation using the fully conditional specification method to impute missing covariates, in a sensitivity analysis. Parameter estimates did not change substantially (Supplementary Tables 1 and 2). Participants reporting food worry had a 2.02 greater odds of endorsing anxiety symptoms (95% CI: 1.50- 2.73, *p* < 0.0001) and a 1.74 times greater odds of reporting depression symptoms (95% CI: 1.2902.35, *p* = 0.0003).

## Discussion

There has been a paucity of literature researching the relationship between mental health and food worry during the COVID-19 pandemic. Our study demonstrates that food worry is associated with increased risk for depression and anxiety symptoms, after controlling for important factors such as pre-existing mental health conditions, education, and income.

We observed an association between food worry and poor mental health among Indigenous participants in univariate analysis but not in the multivariate analysis after adjusting for socio-economic factors such as income and education. This highlights the important contribution of socio-economic disparities on poor health outcomes affecting Indigenous Peoples. This is consistent with previous literature suggesting that socioeconomic variables are important in explaining disparities in health among Indigenous and non-Indigenous people in Canada. Park et al. [24] showed that the risk for avoidable mortality was reduced by 32 – 47% in Indigenous participants when controlling for education and income. The impacts of colonialism, ongoing structural discrimination and continued disregard for Indigenous autonomy has subjected many Indigenous People to racism, poverty, poor education, and residential instability, which all contribute to poor mental health [32, 33]. Structural discrimination and trauma impact Indigenous communities in ways that have been linked to high prevalence of depression and suicide [32]. Additionally, Arriagada and colleagues noted that Indigenous People in urban areas may be more vulnerable to socioeconomic challenges during the pandemic, due to existing disparities prior to COVID-19 [34].

Our findings showed an increased prevalence of food worry during the pandemic, from 7.8 to 14.8%, which is consistent with Canadian national surveys [2]. In addition, food worry during the pandemic was associated with both depression and anxiety symptoms, which is also consistent with some studies conducted during COVID-19. Fang et al. [16] found that during the pandemic, food insecurity in low-income American participants was associated with a higher risk of anxiety (OR 3.57; 95% CI 3.01 – 4.23) and depression (OR 3.53; 95% CI 2.99 – 4.17) [18]. Although these odds ratios are higher than in our study, we did not solely survey low-income participants, and used a Canadian cohort that may have experienced the pandemic differently from the American participants. For example, there was relatively low prevalence of COVID-19 in the Kingston area in comparison to much of the United States. Additionally, government-funded measures in Canada ensured the availability of financial support. For example, the Canada Emergency Response Benefit helped to mitigate income loss for employed and self-employed Canadians who were unable to work due to pandemic related shutdowns, by providing $2000 for a 4-week period. Wolfson et al. found that very low food security was associated with increased risk of depression (OR 7.49; 95% CI 5.52–10.80), anxiety (OR 6.19; 95% CI 4.51–8.51), and high perceived stress (OR 10.91; 95% CI 7.78–15.30) compared to being food secure during the pandemic [19]. In contrast to our study, Wolfson et al. specifically examined the impact of very low food security. The focus of our study was food worry, which is characterized by milder or marginal forms of food insecurity, and which differs from more severe food insecurity. The difference in severity of food insecurity may explain the stronger association between food insecurity and poor mental health found by Wolfson et al.

Our findings differed from with those of McAuliffe et al., [7] who found that participants with food worry were more likely to feel anxious or worried (OR 1.36, 95% CI: 1.08 – 1.71) but were not more likely to feel depressed (OR 1.25, 95% CI: 0.97 – 1.61) [7]. This study did not control for pre-pandemic food worry, which was included in our analysis. In comparison to our study, McAuliffe and colleagues utilized a different avenue of data collection by distributing their online survey via the national polling vendor Maru/Matchbox, which randomly invites panel members (a total of 125,000 Canadian adults) to participate based on stratifications based on sociodemographic characteristics. The survey data collection occurred over a shorter duration compared to our study, from May 14–29, 2020. These dates corresponded with the loosening of restrictions that were initially implemented at the beginning of the pandemic. Our study collected responses over an 8 month period, beginning prior to the reopening after lockdown and including the time period in Fall 2020 when case numbers once again continued to increase. There were also differences between the study cohorts which may contribute to the contrasting results: the McAuliffe cohort has a considerably higher prevalence of participants who had a household income greater than $100,000 than our cohort (42.8 vs. 13.63%). Additionally, the proportion of Indigenous were more than three times higher in our cohort compared to the McAuliffe study (11.14 vs. 3.0%),

The association between food insecurity and poorer mental health outcomes had been described before the COVID-19 pandemic. Nagata and colleagues found that even when adjusting for socio-demographic characteristics, food-insecure young adults were at increased risk of depression (OR 1.67, 95% CI 1.16–1.87) and anxiety or panic disorder (OR 2.76, 95% CI 2.14 – 3.55) [10]. Recently, Shafiee et al. found that household food insecurity predicted depressive symptoms after adjusting for other known risk factors (OR 2.87, 95% CI 2.33–2.55) in the 2015–2016 Canadian Community Health Survey [11]. More specific to the role of marginal food insecurity and food worry, past studies have supported the association between marginal food insecurity and poorer mental health [35–37]. Kolovos and colleagues found that participants with mild food insecurity were more likely to have depression than participants with moderate or severe food insecurity (OR 1.47, 95% CI 1.27–1.71) [36]. Similarly, in a study involving Canadian young adults, marginal food insecurity was associated with an increased risk of fair or poor mental health (OR 1.46, 95% CI 1.28–1.68), major depression (OR 1.96, 95% CI 1.59–2.40), and suicidal ideation (OR 1.77, 95% CI 1.21–2.60) [37].

The consistent evidence demonstrating the association between food worry and poor mental health, and of worsening food insecurity and mental health during the pandemic have important policy and practice implications. Food insecurity is closely tied with income [38, 39], and prior work has explored reducing income insecurity as a means of addressing food insecurity [40, 41]. Thus, our findings support the argument advanced by authors on the importance of robust income support policies, such as a Universal Basic Income, which may have important health benefits. In clinical practice, the strong relationship between food worry and poor mental health underscores the value of health care provider screening for social determinants of health such as food worry during primary care visits. Multiple health professional associations are recommending that physicians screen patients for social determinants of health such as food insecurity and refer those affected to community supports [42–44]. Screening is feasible and effective when conducted in the context of established, trusting relationships in which health professionals work to reduce stigma related to food insecurity [45]. Additionally, supports for behavioural strategies such as meal planning may be beneficial for food insecure households [46].

Based on the high prevalence of food worry among Indigenous respondents, driven by lower income and education among the Indigenous respondents, our findings show a need to target the socioeconomic inequity between Indigenous and non-Indigenous people. At the same time, developing culturally appropriate approaches to addressing food worry in Indigenous communities is important. Building on research that has explored Indigenous Food Sovereignty initiatives as a culturally appropriate approach to enhance wellbeing (i.e. mental health and life satisfaction) and mitigate food insecurity [47, 48], our findings support the importance of adequate funding for community driven solutions to mitigate food worry and contribute to Indigenous Food Sovereignty.

There are several limitations of the study. Participant reports of pre-pandemic experiences are susceptible to recall bias. Due to the cross-sectional design, we are unable to determine causality or the direction of the relationship between food worry and poorer mental health. While the analyses accounted for pre-existing mental health condition, we did not have baseline measures for the GAD-2 or PHQ-2 pre-pandemic. Finally, the representativeness of the cohort is limited by using a convenience sample. The study cohort was primarily young, female, and of European descent, although did include a significant number of Indigenous participants. However, our sample did include participants with a range of incomes, and levels of education.

As the COVID-19 pandemic continues to affect daily life globally, future research is needed to understand longitudinal trends of food worry, and anxiety and depression. In addition, evaluations using qualitative data will add additional insights into the experiences of food worry and poor mental health.

## Conclusion

Our study found increased food worry during COVID-19 from pre-pandemic levels, and that food worry was associated with an increased risk of depression and anxiety symptoms. As the COVID-19 pandemic continues, there is an opportunity to utilize findings from the early months of the pandemic and its consequences for Canadians to address mental health needs, as well as mitigate effects from future waves of the pandemic. The findings from this study have implications for clinical practice, public policy, and community resources.

## Supplementary Information

**Additional file 1. Supplementary Table1.** Multivariate logistic regression models of the association between Foodworry and symptoms of anxiety (GAD-2 ≥ 3) with multiple imputation. **SupplementaryTable 2.** Multivariate logistic regression models of the association betweenFood worry and symptoms of depression (PHQ-2 ≥ 3) with multiple imputation.

## Data Availability

The datasets generated and/or analysed during the current study are not publicly available to ensure data integrity and avoid scientific overlap between projects but are available from the corresponding author on reasonable request.

## Abbreviations

OCAP®: Ownership, Control, Access, Possession
GAD-2: Generalized Anxiety Disorder-2
PHQ-2: Personalized Health Questionnaire-2

## References

[1] Hetrick RL, Rodrigo OD, Bocchini CE (2020). Addressing Pandemic-Intensified Food Insecurity. Pediatrics.

[2] Polsky JY, Gilmour H (2020). Food insecurity and mental health during the COVID-19 pandemic. Health Rep.

[3] Willows N, Veugelers P, Raine K, Kuhle S (2011). Associations between household food insecurity and health outcomes in the aboriginal population (excluding reserves). Health Rep.

[4] Levkoe CZ, McLaughlin J, Strutt C (2021). Mobilizing networks and relationships through indigenous food sovereignty: the indigenous food circle’s response to the COVID-19 pandemic in Northwestern Ontario. Front Commun.

[5] Tarasuk V, Mitchell A. 2020. Household food insecurity in Canada, 2017–18. Toronto.

[6] Canadian Community Health Survey. The Household Food Security Survey Module [Available from: https://www.canada.ca/en/health-canada/services/food-nutrition/food-nutrition-surveillance/health-nutrition-surveys/canadian-community-health-survey-cchs/household-food-insecurity-canada-overview/household-food-security-survey-module-hfssm-health-nutrition-surveys-health-canada.html.

[7] McAuliffe C, Daly Z, Black J, Pumarino J, Gadermann A, Slemon A (2021). Examining the associations between food worry and mental health during the early months of the COVID-19 pandemic in Canada. Can J Public Health.

[8] Coleman-Jensen AJ (2010). US food insecurity status: toward a refined definition. Soc Indic Res.

[9] Darling KE, Fahrenkamp AJ, Wilson SM, D’Auria AL, Sato AF (2017). Physical and mental health outcomes associated with prior food insecurity among young adults. J Health Psychol.

[10] Nagata JM, Palar K, Gooding HC, Garber AK, Whittle HJ, Bibbins-Domingo K (2019). Food insecurity is associated with poorer mental health and sleep outcomes in young adults. J Adolesc Health.

[11] Shafiee M, Vatanparast H, Janzen B, Serahati S, Keshavarz P, Jandaghi P (2021). Household food insecurity is associated with depressive symptoms in the Canadian adult population. J Affect Disord.

[12] Muldoon KA, Duff PK, Fielden S, Anema A (2013). Food insufficiency is associated with psychiatric morbidity in a nationally representative study of mental illness among food insecure Canadians. Soc Psychiatry Psychiatr Epidemiol.

[13] Cook JT, Black M, Chilton M, Cutts D, de EttingerCuba S, Heeren TC (2013). Are food insecurity's health impacts underestimated in the US population? Marginal food security also predicts adverse health outcomes in young US children and mothers. Adv Nutr.

[14] Jessiman-Perreault G, McIntyre L (2017). The household food insecurity gradient and potential reductions in adverse population mental health outcomes in Canadian adults. SSM-Population Health.

[15] Jessiman-Perreault G, McIntyre L (2019). Household food insecurity narrows the sex gap in five adverse mental health outcomes among Canadian adults. Int J Environ Res Public Health.

[16] Tarasuk V, Fafard St-Germain A-A, Mitchell A (2019). Geographic and socio-demographic predictors of household food insecurity in Canada, 2011–12. BMC Public Health.

[17] Sareen J, Afifi TO, McMillan KA, Asmundson GJG (2011). Relationship between household income and mental disorders: findings from a population-based longitudinal study. Arch Gen Psychiatry.

[18] Fang D, Thomsen MR, Nayga RM (2021). The association between food insecurity and mental health during the COVID-19 pandemic. BMC Public Health.

[19] Wolfson JA, Leung CW (2020). Food insecurity and COVID-19: disparities in early effects for US adults. Nutrients.

[20] OCAP: First Nations Information Governance Centre; 2021 [Available from: https://fnigc.ca/ocap-training.

[21] Gilbody S, Richards D, Brealey S, Hewitt C (2007). Screening for depression in medical settings with the Patient Health Questionnaire (PHQ): a diagnostic meta-analysis. J Gen Intern Med.

[22] Löwe B, Kroenke K, Gräfe K (2005). Detecting and monitoring depression with a two-item questionnaire (PHQ-2). J Psychosom Res.

[23] Kroenke K, Spitzer RL, Williams JB (2003). The patient health questionnaire-2: validity of a two-item depression screener. Med Care.

[24] Gelaye B, Wilson I, Berhane HY, Deyessa N, Bahretibeb Y, Wondimagegn D (2016). Diagnostic validity of the Patient Health Questionnaire-2 (PHQ-2) among Ethiopian adults. Compr Psychiatry.

[25] Monahan PO, Shacham E, Reece M, Kroenke K, Ong'or WO, Omollo O (2009). Validity/reliability of PHQ-9 and PHQ-2 depression scales among adults living with HIV/AIDS in western Kenya. J Gen Intern Med.

[26] Tsai FJ, Huang YH, Liu HC, Huang KY, Huang YH, Liu SI (2014). Patient health questionnaire for school-based depression screening among Chinese adolescents. Pediatrics.

[27] Zhang YL, Liang W, Chen ZM, Zhang HM, Zhang JH, Weng XQ (2013). Validity and reliability of patient health questionnaire-9 and patient health questionnaire-2 to screen for depression among college students in China. Asia Pac Psychiatry.

[28] Skapinakis P (2007). The 2-item generalized anxiety disorder scale had high sensitivity and specificity for detecting GAD in primary care. Evid Based Med.

[29] Plummer F, Manea L, Trepel D, McMillan D (2016). Screening for anxiety disorders with the GAD-7 and GAD-2: a systematic review and diagnostic metaanalysis. Gen Hosp Psychiatry.

[30] Staples LG, Dear BF, Gandy M, Fogliati V, Fogliati R, Karin E (2019). Psychometric properties and clinical utility of brief measures of depression, anxiety, and general distress: the PHQ-2, GAD-2, and K-6. Gen Hosp Psychiatry.

[31] SAS OnDemand for Academics. SAS Institute Inc. 2021. https://www.sas.com/en_ca/software/on-demand-for-academics.html.

[32] King M, Smith A, Gracey M (2009). Indigenous health part 2: the underlying causes of the health gap. The lancet.

[33] Paradies YC, Cunningham J (2012). The DRUID study: racism and self-assessed health status in an indigenous population. BMC Public Health.

[34] Arriagada P, Hahmann T, O'Donnell V. 2020. Indigenous people in urban areas: Vulnerabilities to the socioeconomic impacts of COVID-19. Statistics Canada= Statistique Canada.

[35] Jones AD (2017). Food insecurity and mental health status: a global analysis of 149 countries. Am J Prev Med.

[36] Kolovos S, Zavala GA, Leijen AS, Melgar-Quiñonez H, van Tulder M (2020). Household food insecurity is associated with depressive symptoms: results from a Mexican population-based survey. Food Security.

[37] Men F, Elgar FJ, Tarasuk V. Food insecurity is associated with mental health problems among Canadian youth. J Epidemiol Community Health. 2021;75(8):741–8. 10.1136/jech-2020-216149. 10.1136/jech-2020-216149 33579754

[38] Loopstra R, Tarasuk V (2015). Food bank usage is a poor indicator of food insecurity: Insights from Canada. Soc Policy Soc.

[39] Tarasuk V, Vogt J (2009). Household food insecurity in Ontario. Can J Public Health.

[40] Emery J, Fleisch V, McIntyre L. How a guaranteed annual income could put food banks out of business. SPP Res Paper. 2013;(6–37):1–21. 10.11575/sppp.v6i0.42452.

[41] McIntyre L, Dutton DJ, Kwok C, Emery JH (2016). Reduction of food insecurity among low-income Canadian seniors as a likely impact of a guaranteed annual income. Can Public Policy.

[42] College of Family Physicians of Canada (2017). Poverty: A Clinical Tool for Primary Care Providers.

[43] American Academy of Family Physicians. Social Determinants of Health Policy [Available from: https://www.aafp.org/about/policies/all/social-determinants.html.

[44] Hagan JF, Shaw JS, Duncan PM, eds. Bright Futures: Guidelines for Health Supervision of Infants, Children, and Adolescents. 4th ed. Elk Grove Village, IL: American Academy of Pediatrics; 2017. https://reader.aappublications.org/bright-futures-guidelines-for-health-supervision-of-infants-children-and-adolescents-4th-ed/3.

[45] Runkle NK, Nelson DA (2021). The silence of food insecurity: disconnections between primary care and community organizations. J Patient Cent Res Rev.

[46] Fiese BH, Gundersen C, Koester B, Jones B (2016). Family chaos and lack of mealtime planning are associated with food insecurity in low-income households. Econ Hum Biol.

[47] Blanchet R, Batal M, Johnson-Down L, Johnson S, Willows N (2021). An Indigenous food sovereignty initiative is positively associated with well-being and cultural connectedness in a survey of Syilx Okanagan adults in British Columbia. Can BMC Public Health.

[48] Cidro J, Adekunle B, Peters E, Martens T (2015). Beyond food security: understanding access to cultural food for urban indigenous people in Winnipeg as Indigenous food sovereignty. Can J Urban Res.

