# Supplementary material for: Food worry and mental health outcomes during the COVID-19 pandemic
Source: BMC Public Health. 2022 May 17;22:994. doi: 10.1186/s12889-022-13410-7 (PMC9111947; doi:10.1186/s12889-022-13410-7)
Supplement: Supplementary file 1 — Additional file 1. Supplementary Table1. Multivariate logistic regression models of the association between Foodworry and symptoms of anxiety (GAD-2 ≥ 3) with multiple imputation. SupplementaryTable 2. Multivariate logistic regression models of the association betweenFood worry and symptoms of depression (PHQ-2 ≥ 3) with multiple imputation. [file 12889_2022_13410_MOESM1_ESM.docx]

**Supplementary Table 1. Multivariate logistic regression models of the association between Food worry and symptoms of anxiety (GAD-2 ≥ 3) with multiple imputation**

| **Variable** | **OR (95% CI)** | **p** |
| --- | --- | --- |
| Current food worry  No current food worry | 2.02 (1.50-2.73)  Reference | <0.001 |
| Past food worry  No past food worry | 1.27 (0.86- 1.90)  Reference | 0.23 |
| **Gender**  Female  Male  Non-binary* | 1.73 (1.22- 2.46)  Reference  1.54 (0.63- 2.79) | 0.0023  0.34 |
| **Ethnicity**  Indigenous  Asian, Middle Eastern, African, Latin American  European | 1.08 (0.80- 1.46)  1.05 (0.76- 1.46)  Reference | 0.63  0.75 |
| **Age**  16 – 34 years old  35 – 49 years old  50 – 64 years old  ≥65 years old | 5.95 (3.30- 10.72)  4.71 (2.59- 8.57)  3.20 (1.715.98)  Reference | <0.0001  <0.0001  0.0003 |
| **Household income**  <$30,000  $30,000 – $79,999  $80,000 – $149,000  ≥$150,000 | 1.44 (1.01- 2.04)  0.92 (0.65- 1.29)  0.99 (0.71- 1.36)  Reference | 0.04  0.62  0.93 |
| **Education****  High school or less  College  University | 1.41 (1.10 -1.81)  1.18 (0.91 -1.52)  Reference | 0.006  0.20 |
| **Pre-existing mental health condition**  Yes  No | 3.47 (2.82-4.26)  Reference | <0.0001 |

**Supplementary Table 2. Multivariate logistic regression models of the association between Food worry and symptoms of depression (PHQ-2 ≥ 3) with multiple imputation**

| **Variable** | **OR (95% CI)** | **p** |
| --- | --- | --- |
| Current food worry  No current food worry | 1.74 (1.29 - 2.35)  Reference | 0.0003 |
| Past food worry  No past food worry | 1.24 (0.84 - 1.83)  Reference | 0.29 |
| **Gender**  Female  Male  Non-binary* | 1.34 (0.94 - 1.92)  Reference  1.28 (0.52 - 3.13) | 0.11  0.59 |
| **Ethnicity**  Indigenous  Asian, Middle Eastern, African, Latin American  European | 1.17 (0.85 - 1.62)  1.14 (0.80 - 1.64)  Reference | 0.33  0.46 |
| **Age**  16 – 34 years old  35 – 49 years old  50 – 64 years old  ≥65 years old | 1.53 (0.97 - 2.42)  1.23 (0.77 - 1.98)  1.19 (0.72 - 1.97)  Reference | 0.07  0.39  0.50 |
| **Household income**  <$30,000  $30,000 – $79,999  $80,000 – $149,000  ≥$150,000 | 1.54 (1.05 - 2.26)  1.05 (0.72 - 1.53)  1.11 (0.78 - 1.59)  Reference | 0.03  0.80  0.57 |
| **Education****  High school or less  College  University | 1.73 (1.34 - 2.23)  1.32 (1.02 - 1.73)  Reference | <0.0001  0.04 |
| **Pre-existing mental health condition**  Yes  No | 3.83 (3.08 - 4.77)  Reference | <0.0001 |
